# Pressure-volume curves of fine roots reveal intraspecific variation across different elevations in a subalpine forest

**DOI:** 10.1007/s10265-025-01618-8

**Published:** 2025-02-08

**Authors:** Taiga Masumoto, Yuki Hashimoto, Takumi Ito, Koichi Takahashi, Naoki Makita

**Affiliations:** Faculty of Science, Shinshu University, 3-1-1 Asahi, Matsumoto, Nagano, 390-8621 Japan

**Keywords:** Capacitance, Mature trees, Nitrogen content, Root tissue density, Turgor loss point, Water conservation

## Abstract

Water conservation in fine roots can be important for the adaptation of trees to cold, nutrient-poor ecosystems. Although pressure–volume (p-v) curve traits are commonly used to assess leaf water conservation, little is known about their intraspecific variation in fine roots and their association with root functional traits, such as morphology and chemistry. Here, we aimed to determine the p-v curve traits of *Betula ermanii* and *Abies mariesii* fine roots at 2,000 and 2,500 m elevations and explore their intraspecific variation with root morphological and chemical traits in a subalpine forest. Turgor loss point (π_tlp_), relative water content at π_tlp_, osmotic potential at full hydration, and capacitance at full turgor (C_ft_) were evaluated as p-v curve traits. Additionally, root diameter, specific root length, and root tissue density (RTD) were assessed as morphological traits, and nitrogen (N) content was measured as a chemical trait. For *A mariesii* roots, the C_ft_ was lower, and π_tlp_ was more negative at 2,500 m than at 2,000 m. The p-v curve traits of *B ermanii* roots remained unchanged with elevation. There were strong correlations between RTD and π_tlp_ and between N content and π_tlp_ and C_ft_, especially for *A. mariesii*. These results indicated *A. mariesii* adjusted p-v curve traits with RTD and N content and achieved water conservation in fine roots at higher elevations. The p-v curve traits, particularly π_tlp_ and C_ft_, reflected diverse tree strategies for environmental acclimation with fine-root carbon economy. Our findings highlighted the importance of adjusting water relation traits for acclimation to cold and nutrient-poor subalpine regions, particularly for evergreen coniferous species. The p-v curve traits revealed diverse fine-root water relation traits as a basis for water conservation capacity by preserving root function under stress conditions and enabling prolonged resource acquisition in a subalpine forest.

**Supplementary Information:**

The online version contains supplementary material available at 10.1007/s10265-025-01618-8.

## Introduction

Fine roots (< 2 mm in diameter) contribute to tree performance by acquiring water and nutrient from the soil (Freschet et al. 2021; Ito et al. 2023; Masumoto et al. 2022; McCormack et al. 2015). In forest ecosystems, soil environments change drastically throughout the year and even within the day (McCormack et al. 2014; Sugai et al. 2024). Therefore, the abilities of trees to maintain and enhance their performance in forest ecosystems may also depend on a “conservation strategy” in the fine roots for preserving function under stress conditions and/or achieving a longer resource acquisition (Bergmann et al. 2020; Roumet et al. 2016; Weemstra et al. 2020). Because the lifespan of roots is a good reflection of a conservation strategy, previous studies have mainly focused on lifespan-related traits (Bergmann et al. 2020; Weemstra et al. 2020). However, a root dysfunction caused by a reduction in root physiological activity likely occurs before root death. In particular, water loss from root cells causes cell shrinkage and reduces contact between the roots and soil, thereby decreasing water flow at the soil–root interface (Cuneo et al. 2016; North and Nobel 1997). Moreover, water loss from the cells causes turgor loss, which greatly impacts growth and physiological activity (Bartlett et al. 2012; McDowell et al. 2008). Therefore, the assessment of water conservation in the fine roots is important for understanding the tree strategy to grow and survive by preserving root function under stress conditions and/or achieving a longer resource acquisition from the soil. Here, root water conservation is defined as the strategy for reducing water loss from cells and preserving contact at the soil–root interface under stress conditions like drought. An accurate evaluation of the water conservation for fine roots could benefit from an assessment of intraspecific trait variation, which reflects the capacity of plants to adapt to soil heterogeneity or climate change (Laughlin and Messier 2015).

It is known that the root water conservation has been used for maintaining plant performance under drought conditions (Cuneo et al. 2016; North and Nobel 1997). North and Nobel (1997) reported that 14-day drought treatment decreased the root-soil contact from 94 to 21% by cell shrinkage in *Agave deserti* and the hydraulic conductivity of overall root-soil pathway reduced about one-fifth. In the case of drought condition, the breaking root-soil interface is also important to reduce water loss from root to soil (Nobel and Cui 1992). On the other hand, water conservation in roots could deepen our understanding not only for drought environment but also for cold environments. A cold environment, like alpine and subalpine, usually has low nutrient availability in soil due to the low decomposition rate (Kӧrner 2012). Trees partially take up soil nutrients with water, because water dissolves large quantities of ions and polar organic metabolites, such as sugars, amino acids, and proteins, which are critical for cell metabolism and growth (Lambers and Oliveira 2019). Therefore, the small contact- area of the root–soil interface can greatly prevent the nutrients uptake, and water conservation may be a potential driver in colder regions than the warmer ecosystems to maintain nutrient balance (Crick and Grime 1987).

Pressure–volume (p-v) curve traits, which characterize the impact of water stress on the turgor and water volume in plant cells (Cheung et al. 1975), are useful for assessing water conservation in leaves together with the diverse way to achieve the water conservation including osmotic and elastic adjustment (Bartlett et al. 2012). With regard to tree roots, Bartlett et al. (2022) reported that the turgor loss point (π_tlp_), osmotic potential at full hydration (π_o_), and capacitance at full turgor (C_ft_) of the eight grapevine rootstocks were significantly reduced by drought treatment, indicating that roots are less susceptible to turgor loss and volumetric shrinkage in drought conditions than in well-watered conditions. Moreover, Aritsara et al. (2022) compared the p-v curve traits of woody species from karst and mangrove forests, two water-stressed habitats, with those of timber and ornamental woody species grown in a well-watered common garden. They observed that the π_tlp_ values were more negative for species growing in karst ecosystems, where dry and wet conditions are more variable than in the mangrove forests and the common garden. These findings indicate that the p-v curve traits of roots are important for tree drought adaptation and can reveal root water conservation (Aritsara et al. 2022; Bartlett et al. 2022). Therefore, evaluation of root p-v curve traits would greatly contribute to understanding root water conservation in subalpine cold regions. However, the intraspecific variations in p-v curve traits of fine roots in relation to complex environmental differences have not been studied to date. Knowledge about the water conservation in the fine roots of trees growing in subalpine cold regions remains lacking, as does that of the ecological significance of p-v curve traits for acclimation to stress environments other than drought.

To clarify the importance of P-V curve characteristics in water conservation, root functional traits are useful for acclimations and ecosystem functions in response to environmental changes (Bergmann et al. 2020; Freschet et al. 2021; Yahara et al. 2019). Root functional traits often indicate species-specific variations along the environmental gradient (Kramer-Walter et al. 2016; Weemstra et al. 2021, 2022). These variations are thought to represent tree acclimations to a wide range of biotic and abiotic factors (Freschet et al. 2017; Weemstra et al. 2021). In particular, root diameter, specific root length (SRL), root tissue density (RTD), and nitrogen (N) content are associated with metabolic activity (Makita et al. 2009, 2012) and lifespan (Bergmann et al. 2020; Liu et al. 2016), and are considered to link tightly with the acquisitive-conservative strategy in fine roots (Bergmann et al. 2020; Roumet et al. 2016). Moreover, RTD and N content of fine roots are related to root anatomical structure (Guo et al. 2008; Kong et al. 2019; Kramer-Walter et al. 2016), which possibly cause the variation of fine root p-v curve traits. P-V curve traits of fine roots have rarely been measured, and how they function in natural forests is still unclear. For example, lower root π_o_, which gives the root greater drought tolerance (Bartlett et al. 2022), may be the acquisitive trait in the root as it could contribute to active water uptake (Schenk et al. 2021). Therefore, the relationship between p-v curve traits and root functional traits can provide the mechanistic reason why and how trees change p-v curve traits of fine roots and contribute to the deep understanding of the role of root p-v curve traits for tree acclimation to environmental changes in subalpine cold regions.

Elevation gradients are valuable systems for examining plant responses to environmental changes (Körner 2007). Several climate and soil variables change along the elevation gradient, and regions at higher altitudes typically have lower temperatures, poorer soil nutrients, and shorter growing seasons (Kӧrner 2012). The morphological, physiological, and chemical characteristics of leaves are typically altered by trees in response to elevation-related environmental changes (e.g., Taneda et al. 2020; Weemstra et al. 2022). Generally, species inhabiting higher altitudes with unfavorable growth conditions imply a strategy of slow return on investment in dry mass (Hikosaka et al. 2002; Read et al. 2014). However, these patterns are known to vary between deciduous broad-leaved trees and evergreen conifers (Hikosaka et al. 2021; Takahashi and Miyajima 2008). For example, Takahashi and Miyajima (2008) reported that at higher elevations, the deciduous broad-leaved tree species *Betula ermanii* Cham. changed its leaf traits toward an “acquisitive strategy” with a higher N content and shorter lifespan, whereas the evergreen coniferous species *Abies mariesii* Mast. changed its leaf traits toward a “conservative strategy” with a lower N content and longer lifespan. This is possibly due to the completely different leaf habits of evergreen and deciduous species; evergreen species typically have a longer leaf lifespan and thicker leaves with higher leaf mass per area optimized to slow the return on investment compared to deciduous species (Reich et al. 1992). For fine roots, coniferous species generally have higher mean root diameter and lower SRL than those of broad-leaved species, suggesting that they could be more resource conservative than broad-leaved species (Liese et al. 2017; Yahara et al. 2019). Considering the link between leaf and root functional traits along the acquisitive-conservative strategy (Liese et al. 2017; Liu et al. 2010), we suspect that the evergreen coniferous roots might equip the water conservation along the elevation conditions compared with deciduous broad-leaved roots with altering the root p-v curve traits. Although the intraspecific variation in p-v curve traits at different elevations has the possibility to differ between broad-leaved and coniferous roots, it remains a challenge to integrate the water conservation and functional traits with phylogenetic contrast in tree fine roots along environmental gradients.

In this study, we aimed to assess intraspecific variation in the p-v curve traits of fine roots using elevational differences in *Betula ermanii*, a deciduous broad-leaved species, and *Abies mariesii*, an evergreen coniferous species, in a subalpine forest. Fine root traits were compared between each species’ upper and lower limits of dominance to determine plant strategy clearly (Körner 2012). We evaluated the relationships between root p-v curve traits and morphological and chemical traits, including mean diameter, SRL, RTD, and root N content, which critically affect tree performance and ecosystem function (Bergmann et al. 2020; Comas and Eissenstat 2004; McCormack et al. 2017). The following three hypotheses were tested as case studies in a subalpine forest. Because subalpine forests typically have low temperatures, poor soil nutrients, and short growing seasons (Kӧrner 2012), (Hypothesis 1) tree fine roots at higher elevations will have p-v curve traits associated with water conservation (i.e., more negative π_tlp_ and π_o_ and lower C_fl._) for maintaining nutrient balance. In particular, (Hypothesis 2) evergreen coniferous roots might show remarkable variation in p-v curve traits along elevations compared to deciduous broad-leaved roots because of their conservative traits in leaves (Reich et al. 1992) and fine roots (Liese et al. 2017; Yahara et al. 2019). Finally, (Hypothesis 3) the root p-v curve traits are strongly related to morphological and chemical traits across elevations, particularly in evergreen coniferous roots, to achieve water conservation at higher elevations.

## Materials and methods

### Study site, species selection, and environmental data

**Fig. 1:**
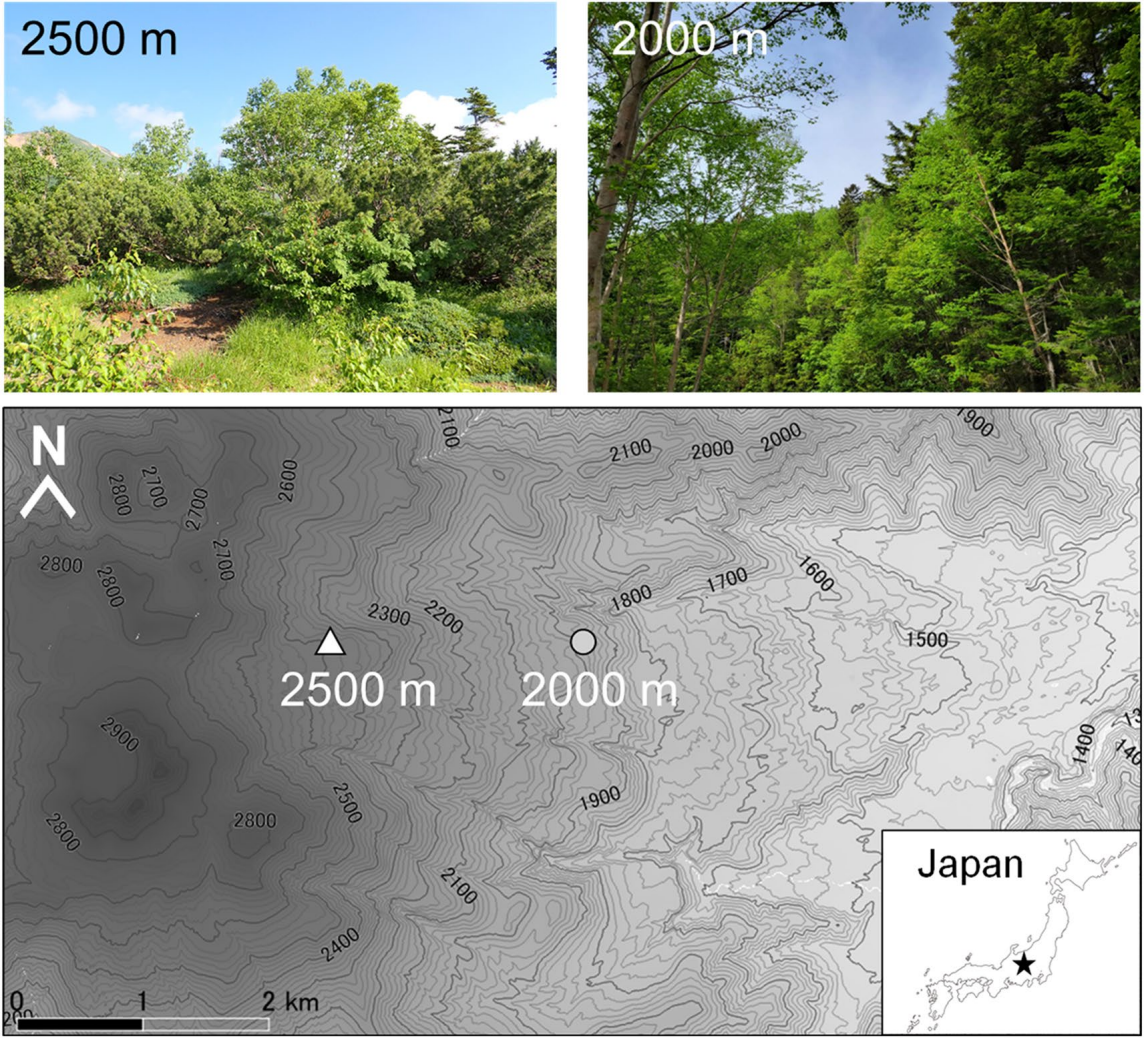
Locations and photographs of each plot established at 2,000 m and 2,500 m on the east slope of Mount Norikura

This study was conducted in the subalpine forest zone on the east slope of Mount Norikura in central Japan. Several studies on intra- and interspecific variations in the above- and below-ground physiological traits of trees have been performed at this site (e.g., Azuma et al. 2024; Hashimoto et al. 2023; Nakamoto et al. 2013). The mean annual precipitation recorded at the Nagawa Weather Station (1,068 m a.s.l.) from 1991 to 2020 was 1,947 mm. We selected two elevations of 2,000 m (N36°06.9746’, E137°35.4656’) and 2,500 m (N36°06.9552’, E137°34.1140’) for the study site (Fig. 1). At 2,000 m a.s.l., the dominant species is *Abies veitchii*, *Tsuga diversifolia*, *Abies mariesii*, and *Betula ermanii* (Miyajima et al. 2007). The 2,500 m a.s.l. point is the upper distribution limit of tall tree species (i.e., alpine treeline; cf., Kӧrner 2012) owing to the strong winds and snow at higher elevations (Takahashi et al. 2012). The tree-growing season is from mid-June to early October at 2,000 m and from mid-July to early September at 2,500 m.

We selected *B. ermanii* and *A. mariesii*, which are common dominant tall species at the two elevations (Miyajima et al. 2007), as target species. *B. ermanii* is a deciduous broad-leaved tree classified as an early successional species, whereas *A. mariesii* is an evergreen conifer classified as a late successional species. The roots of both species are in symbiosis with ectomycorrhizal fungi.

Environmental data at each elevation were monitored from early August to early September 2022 during the growing season. Air temperature was monitored at approximately 1.0 m above the ground using a data logger (LR5001, Hioki, Nagano, Japan). Soil physical properties were monitored on the ground at a depth of 0–15 cm using a data logger (Temperature: RC-5, Elitech Technology Inc., CA, USA, and water potential: DIK-3210 i Tensiometer, Daiki Rika Kogyo Co., Ltd., Osaka, Japan). The mean soil temperature during the growing season was 15.1 °C at 2,000 m, 2.4 °C higher than that at 2,500 m (Table S1). The mean air temperature during the growing season was 15.5 °C at 2,000 m, 2.2 °C higher than that at 2,500 m. The mean soil water potential was increase from − 2.12 to − 1.29 kPa, but differed only slightly between 2,000 and 2,500 m during the study period.

Soil samples were collected in September 2022 from a 50 m × 50 m plot established for root sampling (see the root collection section for details) to determine the soil chemical characteristics at each elevation. We selected six subplots that included the surveyed individuals. The top 10 cm of soil was sampled after removing the fresh litter layer within 150 cm of the surveyed individuals. Each sampling point was at least 10 m apart. The samples were sieved through a 2 mm sieve and measured for soil ammonium content (NH_4_-N) using the Berthelot method (Shand et al. 2008) and nitrate content (NO_3_-N) using the Griess method (Miranda et al. 2001). The total dissolved organic N content (TDN) was determined using the Peroxo Oxidizing Reagent method. Dissolved organic N content (DON) was calculated by subtracting the sum of NH_4_-N and NO_3_-N from TDN. TDN greatly decreased from 89.6 to 60.2 mg kg^− 1^ together with decreasing of dissolved organic nitrogen content from 64.0 to 39.9 mg kg^− 1^ (Table S2).

### Root collection

**Fig. 2:**
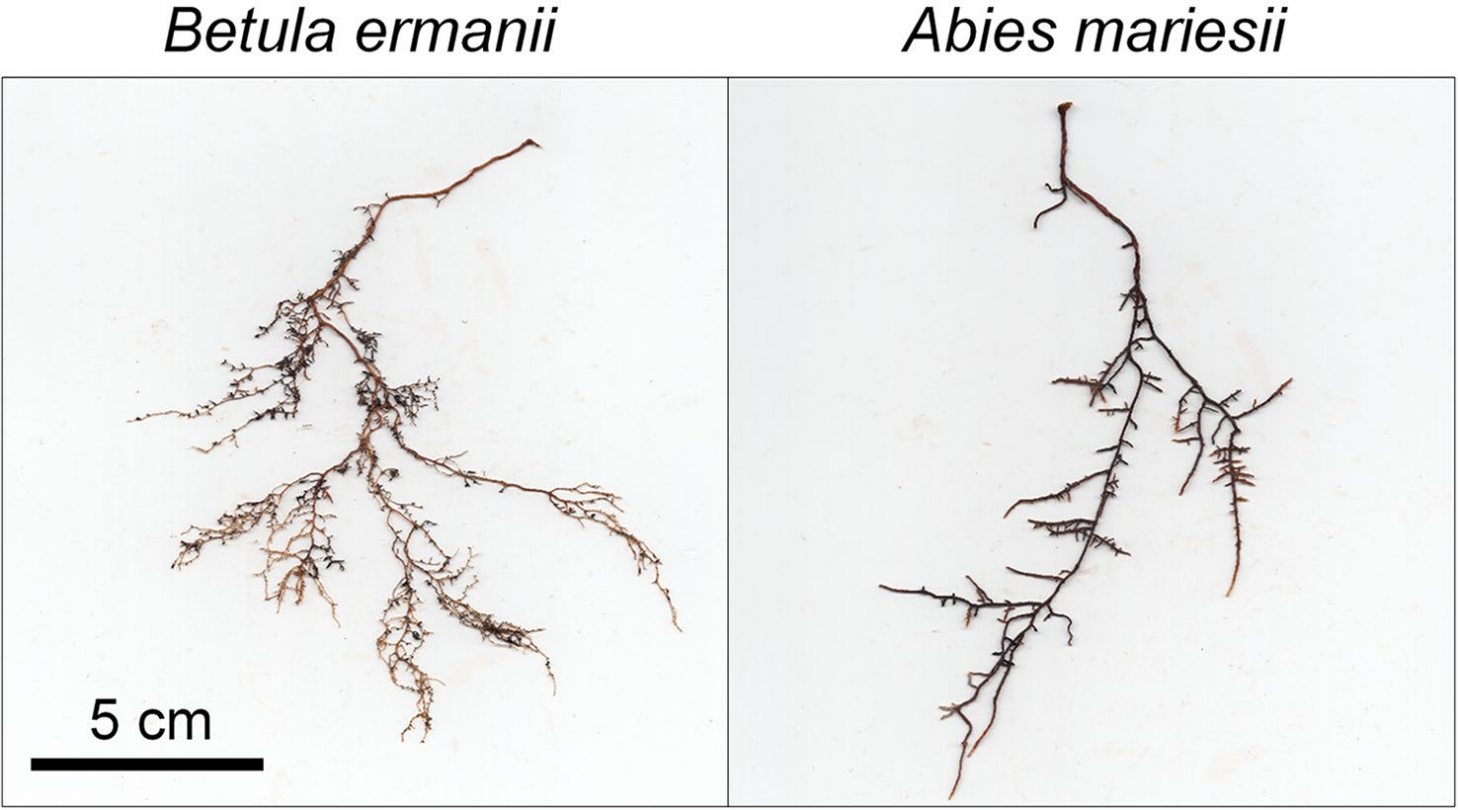
The photographs of intact fine root systems of *Betula ermanii* and *Abies mariesii*

Root sampling was conducted during the growing season from early August to early September 2022. We established a 50 m × 50 m plot at each elevation (Fig. 1) and divided it into 25 subplots with 10 m × 10 m. We selected 11 subplots for sampling from each elevation level based on the following criteria: (1) more than two mature targeted species were present in the subplot, and (2) there was relatively little runoff and erosion of the soil. The stem diameters at approximately 100 cm tree height were 21.5 ± 3.6 cm (mean ± SD) for *B. ermanii* and 38.2 ± 2.2 cm for *A. mariesii* at 2,000 m a.s.l. and 24.3 ± 2.9 cm for *B. ermanii* and 16.2 ± 1.4 cm for *A. mariesii* at 2,500 m a.s.l. The tree heights were 13.1 ± 0.9 m for *B. ermanii* and 18.0 ± 1.1 m for *A. mariesii* at 2,000 m a.s.l. and 6.0 ± 0.2 m for *B. ermanii* and 4.0 ± 0.9 m for *A. mariesii* at 2,500 m a.s.l. Using pruning shears and a shovel, we excavated one to two fine root systems at a depth of 10 cm from the top of the soil, including the organic layer, within 150 cm of each target tree. The diameter, branching pattern, color, and texture of the root bark and epidermis were used to identify the fine root systems of each species according to the method outlined by Yahara et al. (2019) (Fig. 2). We distinguished living from dead roots based on their color and elasticity and carefully collected the root systems based on the following criteria: (1) root systems included the 1st - to 5th orders based on the branching order classification (Pregitzer et al. 2002) and (2) all parts of the root systems were living and intact. Since dead roots or their remnants could interfere with measurements, we paid close attention to selecting the root systems in which all parts were living and intact. All fine root systems were carefully isolated from the soil organic matter and gently washed with tap water and distilled water. Finally, 77 root systems (2 elevations × 2 species × 11 target trees × 1–2 replicates) were collected. Each root system was placed in deionized water (~ 20 °C) and transported to the laboratory within 4 h for further measurements.

### Root pressure–volume curve analyses

To analyze the root pressure-volume (p-v) curve of fine root systems, we modified the Bartlett et al. (2022) pressure chamber method. In the laboratory, the root systems were scanned in grayscale at 600 dpi using a scanner (GT-S650, Epson, Nagano, Japan). Each root system was then rinsed with deionized water and hydrated overnight (< 24 h) in a plastic box filled with deionized water (~ 20 °C). After hydration, each root system was gently wiped dry with a paper towel and enclosed in a double bag (humidified by placing a wet paper towel in the outer bag), for 10 min to allow equilibration of the root water potential (Ψ). The outer bag was removed during the measurements to prevent the evaporation of the paper towel from affecting the mass. We then measured the mass of the root itself using a scale with 0.0001 g precision. Subsequently, the root Ψ was measured with a pressure chamber (Model 600, PMS Inc., Oregon, USA) by observing the cut root surface through a dissecting scope (PEAK wide Stand Microscope 20×, Tokai Sangyo Co., Ltd, Tokyo, Japan) as the chamber was slowly pressurized (~ 0.05 MPa s^–1^) until water emerged. Roots were placed in bags during the Ψ measurement to avoid excessive dehydration. Samples with an initial Ψ value more negative than − 0.1 MPa were excluded from the analysis. To construct the curves, these measurements were repeated 8–16 times per root at approximately 0.05–0.20 MPa intervals. Each root was removed from the bag for dehydration between repeated measurements of root mass and Ψ.

The root systems were dried at 50 °C for more than 48 h, and the dry mass was used to calculate the relative water content (RWC), which is the ratio of water in the root sample at the current state to water in the sample at full hydration. To estimate a turgor loss point, the p-v curve traits were interpolated from these relationships using standard methods (Sack and Pasquet-Kok 2011). The π_tlp_ (MPa) and relative water content at turgor loss point (RWC_tlp_; %) were defined graphically as their respective values at which the relationship between RWC and − 1/Ψ transitioned from curvilinear to linear (Fig.S1). Samples exhibited irregular curves (i.e., did not show a transition point from curvilinear to linear in the relationship between RWC and − 1/Ψ) were excluded from the analysis. The linear relationship between RWC and − 1/Ψ was extrapolated to RWC = 1 to calculate π_o_ (MPa). The C_ft_ (MPa^–1^), defined as the slope from the linear regression of RWC against Ψ above π_tlp_, was calculated as follows:

$$\:{\text{C}}_{\text{f}\text{t}}=\frac{\text{d}\text{R}\text{W}\text{C}}{\text{d}{\uppsi\:}}$$

Six root systems could not be evaluated due to the initial value errors or irregular curves, possibly because of the inclusion of dead roots or their remnants, and were excluded from the analysis. Ultimately, 71 root systems (2 elevations × 2 species × 11 target trees × 1–2 replicates) were analyzed.

### Analyses of root morphological and chemical traits

The total root project area (m^2^), length (m), root volume (calculated from root projected area and length by assuming the root as a cylinder; cm^3^), and mean root diameter (mm) of each root system were analyzed for the morphological traits using WinRHIZO Pro 2013a (Regent Instruments, Quebec, Canada). The SRL (m g^–1^) was calculated by dividing the total root length by the dry mass. The total root volume and dry mass were used to calculate the RTD (g cm^− 3^). The dried root systems were then ground into a fine powder for evaluation of the root N content (mg g^− 1^) using a CN analyzer (Flash EA 1112, Thermo Fisher Scientific, MA, USA).

### Statistical analyses

The mean values of the root p-v curve traits (π_tlp_, RWC_tlp_, C_ft_, and π_o_), morphological traits (diameter, SRL, and RTD), and chemical trait (N content) at 2,000 and 2,500 m were calculated for each species by averaging the means for the target trees (*n* = 11). The Brunner–Munzel test was used to identify elevational differences within species for each root trait (*P* < 0.05) (Brunner and Munzel 2000). Spearman rank correlation test was used to examine key interspecific trait correlations within species (*n* = 22, *P* < 0.05). Principal component analysis (PCA) was used to characterize the fine root traits of *B. ermanii* and *A. mariesii*. Differences in trait syndromes between species were tested using multivariate analysis of variance (MANOVA). All statistical analyses were performed using R version 4.1.2 software (R Core Team 2021).

## Results

### Elevational variation in root p-v curve traits

**Fig. 3:**
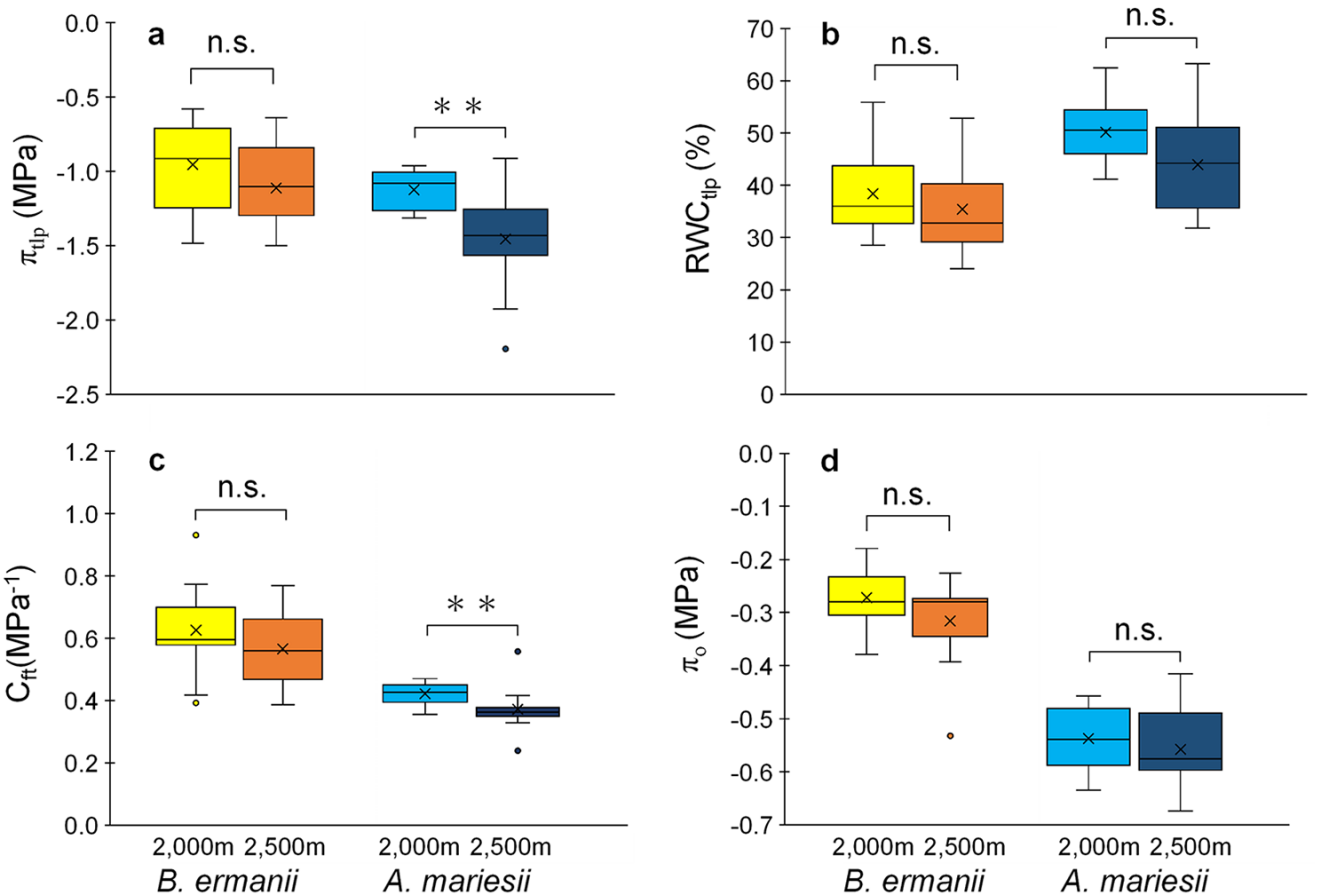
Elevational difference of root pressure-volume curve traits between 2,000 and 2,500 m in *Betula ermanii* and *Abies mariesii*. (**a**) Turgor loss point (π_tlp_). (**b**) Relative water content at turgor loss point (RWC_tlp_). (**c**) Capacitance at full turgor (C_ft_). (**d**) Osmotic potential at full hydration (π_o_). Box plots display median, 25th and 75th percentiles, and minimum and maximum values and points outside the box represent outliers. Cross markers denote the mean values. Statistical significance is indicated by asterisk (Brunner-Munzel test; **, *P* < 0.01; n.s., *P* > 0.05). *n* = 11

The p-v curve traits of the fine roots of mature trees in a natural subalpine forest were measured directly to evaluate their intraspecific variation. Among the four p-v curve traits investigated, π_tlp_ and C_ft_ were the only two that showed species-specific change with elevation (Fig. 3, Table S3). For *B. ermanii*, the π_tlp_ value (mean ± SD) was − 0.95 ± 0.09 MPa at 2,000 m, and − 1.11 ± 0.09 MPa at 2,500 m and there was no significant difference in π_tlp_ (Fig. 3a; *P* > 0.05). In contrast, the π_tlp_ value of *A. mariesii* was − 1.12 ± 0.04 MPa at 2,000 m and decreased significantly to − 1.46 ± 0.10 MPa at 2,500 m. (*P* < 0.01). There were no significant intraspecific differences in RWC_tlp_ among species (Fig. 3b; *P* > 0.05). The C_ft_ value of *B. ermanii* was 0.63 ± 0.05 MPa^− 1^ at 2,000 m and 0.57 ± 0.04 MPa^− 1^ at 2,500 m. There was no significant difference in C_ft_ between the two elevations in *B. ermanii* (Fig. 3c; *P* > 0.05). The C_ft_ value was 0.41 ± 0.01 MPa^− 1^ for *A. mariesii* at 2,000 m and decreased significantly to 0.35 ± 0.03 MPa^− 1^ at 2,500 m (*P* < 0.01). There were no significant intraspecific differences in π_o_ between 2,000 and 2,500 m (Fig. 3d; *P* > 0.05).

### Relationship of C_ft_ and πo to πtlp and RWC_tlp_

**Fig. 4:**
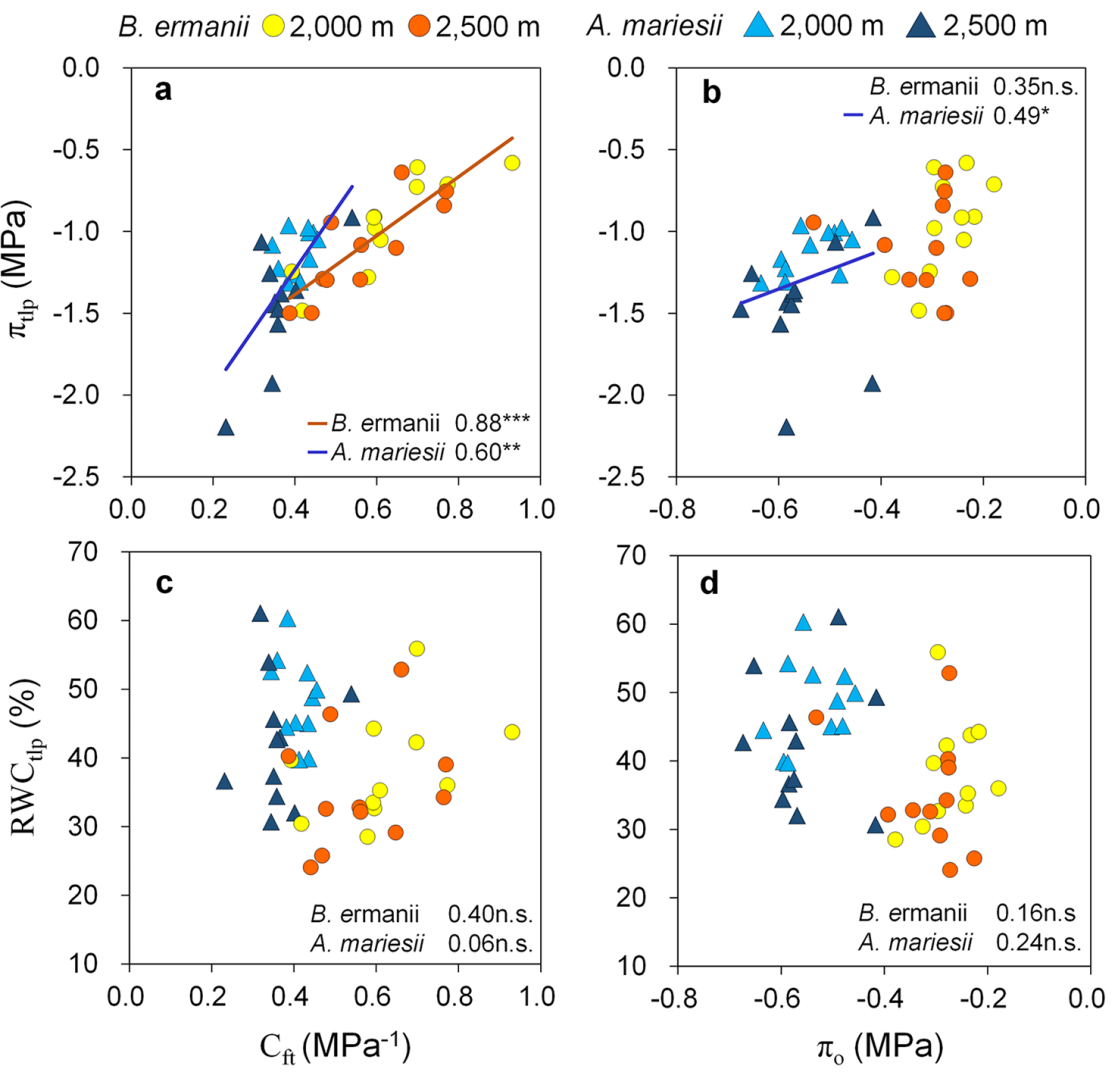
Relationships of turgor loss point (π_tlp_) and relative water content at turgor loss point (RWC_tlp_) with capacitance at full turgor (C_ft_) (**a**, **c**) and osmotic potential at full hydration (π_o_) (**b**, **d**). Regression lines, Spearman rank correlation coefficient within each species are shown (*n* = 22). Statistical significance is indicated by asterisk (***, *P* < 0.001; **, *P* < 0.01; *, *P* < 0.05; n.s., *P* > 0.05)

π_tlp_ correlated positively with C_ft_ for both *B. ermanii* and *A. mariesii* (*B. ermanii*: *r* = 0.88, *P* < 0.001; *A. mariesii*: *r* = 0.60, *P* < 0.01; Fig. 4a), whereas it had positive correlation with π_o_ only in *A. mariesii* (*r* = 0.49, *P* < 0.05; Fig. 4b). The RWC_tlp_ had no relationships with C_ft_ and π_o_ within each species (*P* > 0.05; Fig. 4c, d).

### Elevational variation in root morphological and chemical traits

**Fig. 5:**
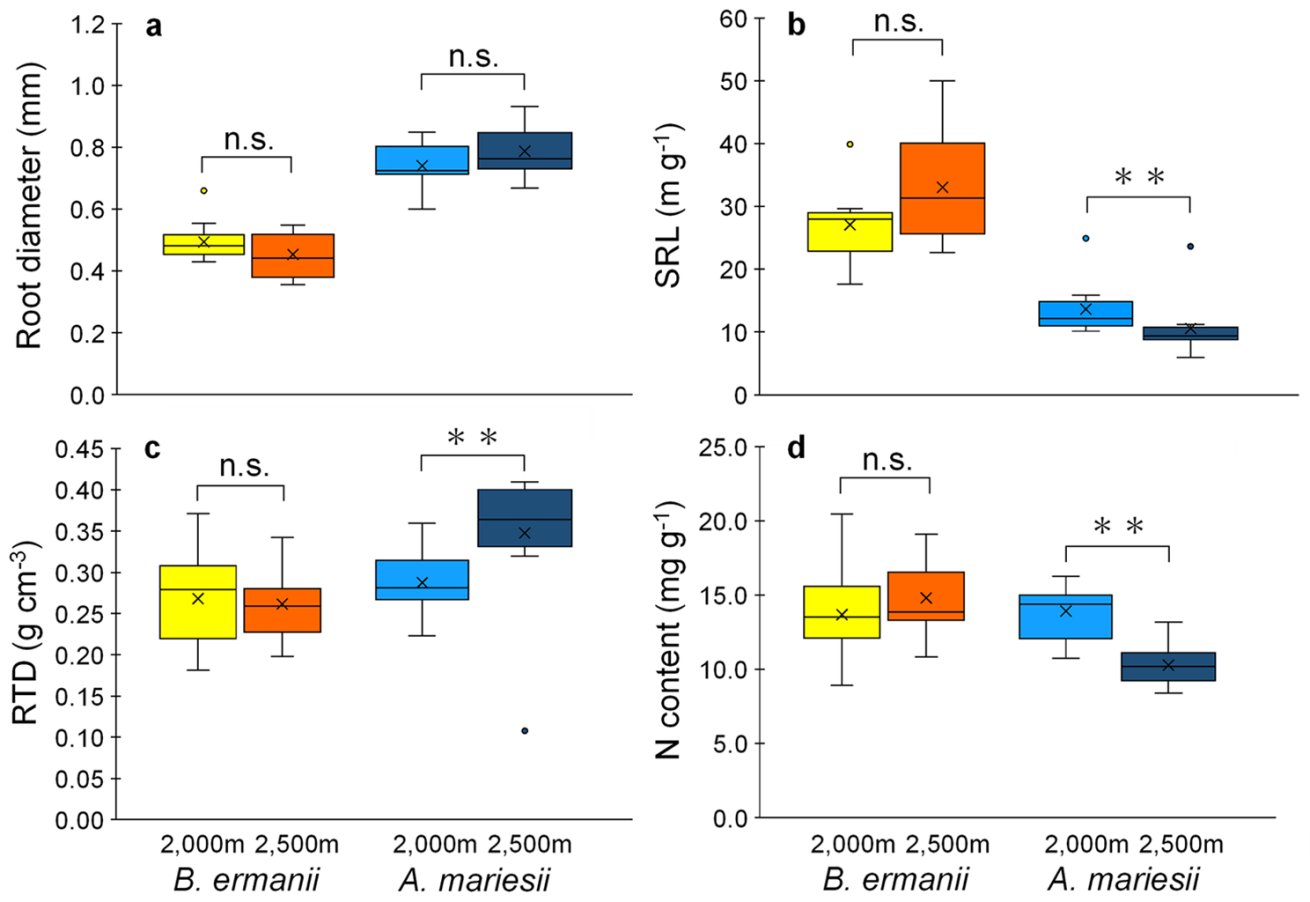
Elevational difference of root morphological and chemical traits between 2,000 and 2,500 m in *Betula ermanii* and *Abies mariesii*. (**a**) Root diameter. (**b**) Specific root length (SRL). (**c**) Root tissue density (RTD). (**d**) Root nitrogen content (N content). Box plots display median, 25th and 75th percentiles, and minimum and maximum values and points outside the box represent outliers. Cross markers denote the mean values. Statistical significance is indicated by asterisk (Brunner-Munzel test; **, *P* < 0.01; n.s., *P* > 0.05). *n* = 11

The elevational differences in root diameter were not significant for either *B. ermanii* or *A. mariesii* (Fig. 5a, Table S3; *P* > 0.05). There was species-dependent variation in RTD, SRL, and N content (Fig. 5b-d, Table S3). The SRL, RTD, and N content did not change significantly with elevation for *B. ermanii* (*P* > 0.05). In contrast, the fine roots of *A. mariesii* showed a significantly higher RTD and lower SRL and N content at 2,500 m than at 2,000 m (*P* < 0.01).

### Relationships between the root p-v curve traits and morphological and chemical traits

**Fig. 6:**
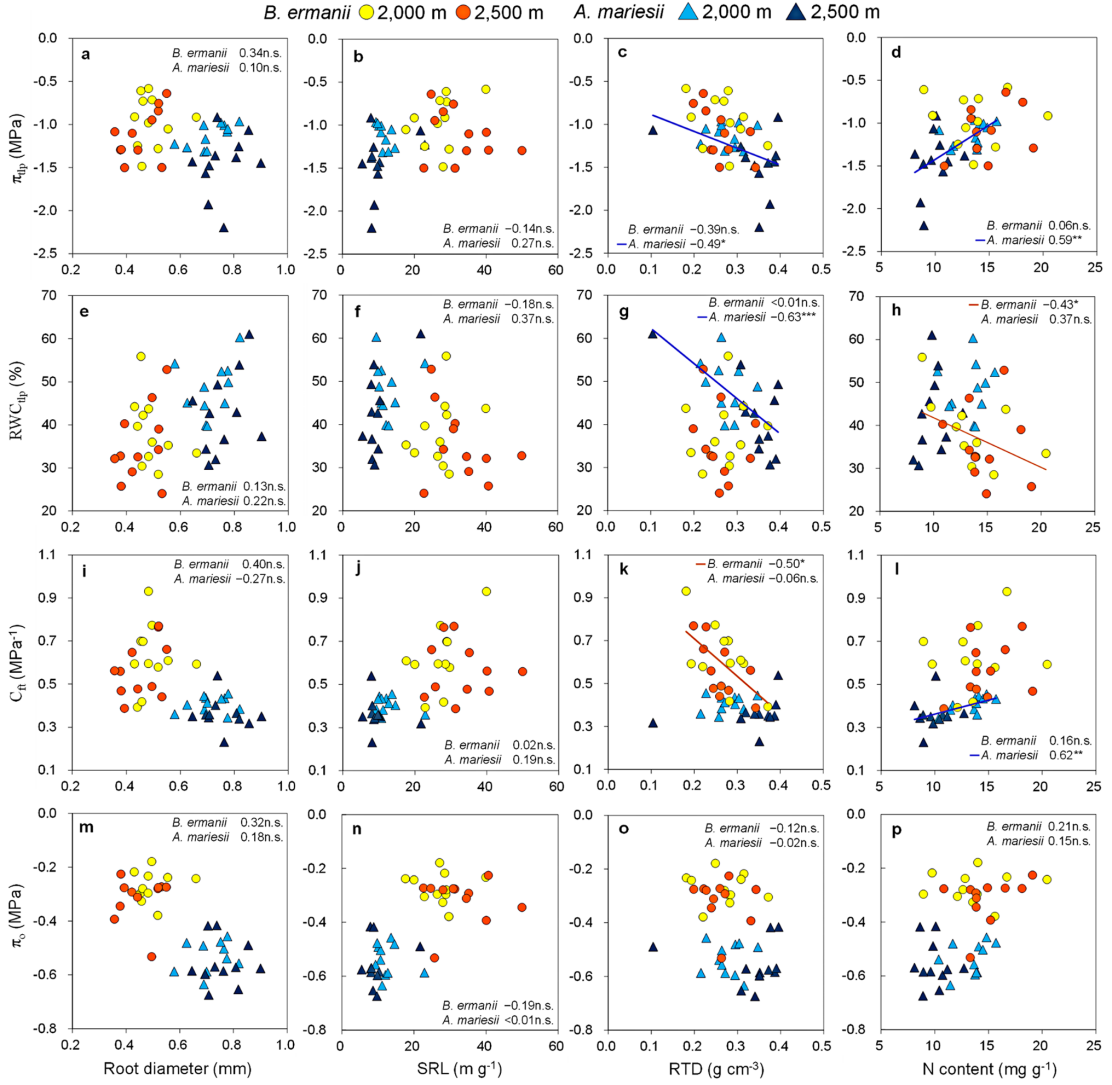
Relationships of turgor loss point (π_tlp_), relative water content at turgor loss point (RWC_tlp_), capacitance at full turgor (C_ft_) and Osmotic potential at full hydration (π_o_) with root diameter (a, e,i, m), specific root length (SRL) (b, f,j, n), root tissue density (RTD) (c, g,k, o) and root nitrogen content (N content) (d, h,l, p). Regression lines, Spearman rank correlation coefficient within each species are shown (*n* = 22). Statistical significance is indicated by asterisk (***, *P* < 0.001; **, *P* < 0.01;*, *P* < 0.05; n.s., *P* > 0.05)

The π_tlp_ showed a negative correlation with RTD only in *A. mariesii* (*r* = − 0.49, *P* < 0.05; Fig. 6c). However, a strong positive correlation was observed between π_tlp_ and the N content only in *A. mariesii* (*r* = 0.59, *P* < 0.01; Fig. 6d). For RWC_tlp_, there was a negative correlation with SRL in *A. mariesii* only (*r* = − 0.63, *P* < 0.001; Fig. 6g) and a negative correlation with N contents in *B. ermanii* only (*r* = − 0.43, *P* < 0.05; Fig. 6h). C_ft_ showed a negative correlation with RTD in *B. ermanii* only (*r* = − 0.50, *P* < 0.05; Fig. 6k) and a positive correlation with the N content in *A. mariesii* only (*r* = 0.62, *P* < 0.01; Fig. 6l). By contrast, π_o_ had no significant relationships with the morphological and chemical traits within species (*P* > 0.05; Fig. 6m-p).

### Overall root characteristics

**Fig. 7:**
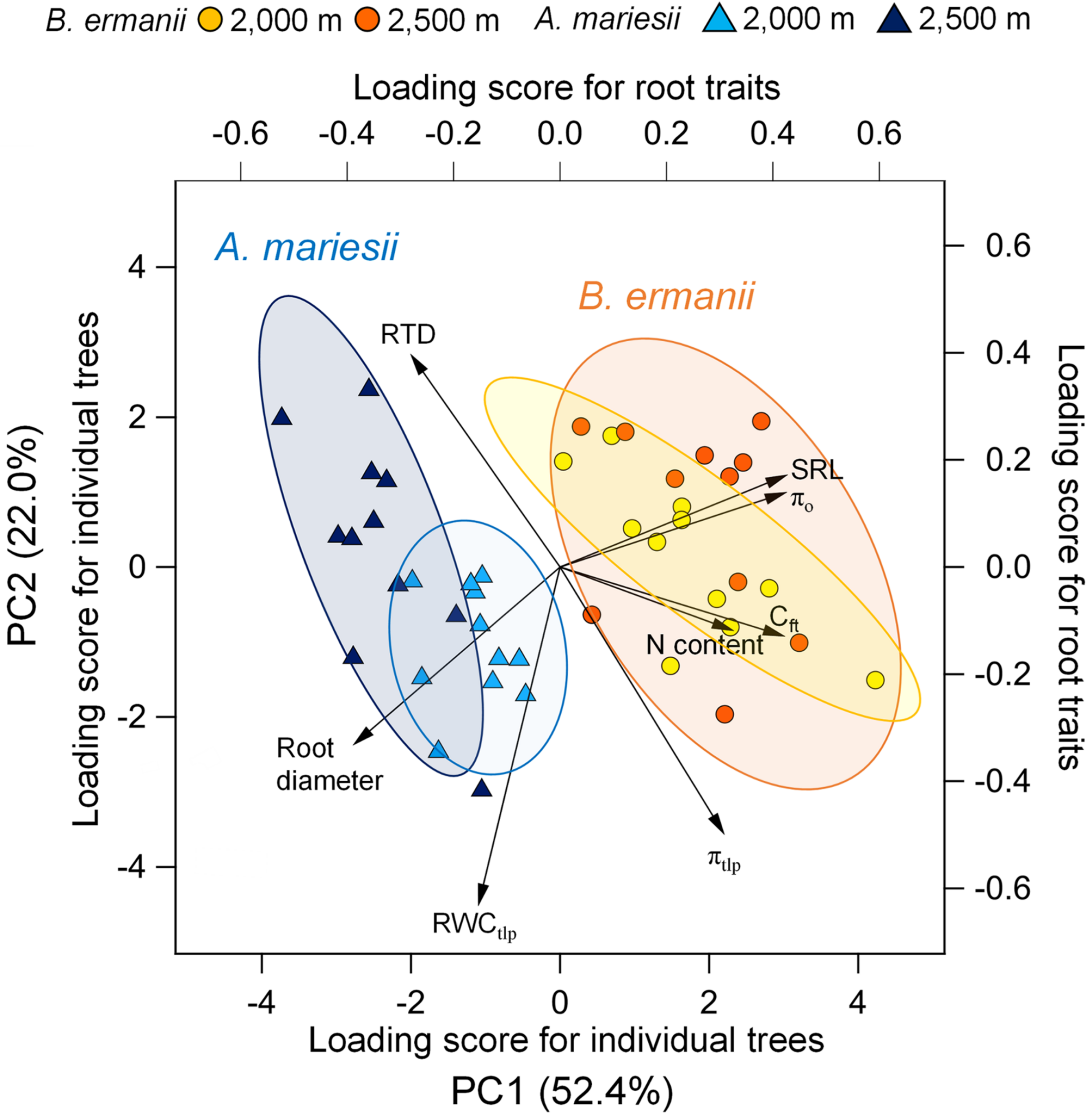
Principal component analysis (PCA) of eight root traits on individual tree level. Abbreviations of each root trait are shown in Table 1. Ellipses represent the 95% confidence interval of *Betula ermanii* and *Abies mariesii* at 2,000 and 2,500 m on the two dimensions. The top and right axis represent load score of eight root traits on two axes. The bottom and left axis represent load score of individuals distribution on the first two dimensions. B. ermanii and *A. mariesii* exhibited significantly different trait coordination tested by MANOVA (*P* < 0.001)

The fine roots of *B. ermanii* and *A. mariesii* were characterized using PCA along with p-v curves and morphological and chemical traits (Fig. 7). The PCA showed that the first principal component (PC1) and second principal component (PC2) accounted for 52.4% and 22.0% of the variation, respectively. Together, these two principal components explained 74.4% of the total variation in all eight root traits. PC1 was aligned with the π_o_, C_ft,_ SRL, and N content. PC2 was strongly aligned with the RWC_tlp_. The PC2 was also weakly aligned with the π_tlp_ and RTD. *B. ermanii* and *A. mariesii* were significantly separated by PC1 (MANOVA; *P* < 0.001), with *B. ermanii* tending to have thinner roots and a higher N content, π_o_, and C_ft_. Particularly, root diameter and SRL strongly explained the difference in fine roots between *B. ermanii* and *A. mariesii* (*P* < 0.001). The variation in root traits at 2,000 m and 2,500 m overlapped in *B. ermanii*, whereas variation in root traits of *A. mariesii* was separated by elevation.

**Table 1:** Abbreviation, unit, and significance of the eight root traits measured in this study

Traits	Abbreviation	Unit	Significance	References
Turgor loss point	π_tlp_	MPa	Water potential when root losses turgor, while root becomes physiologically dysfunctional	Bartlett et al. (2012)
Relative water content at turgor loss point	RWC_tlp_	%	Root hydration at which cells become flaccid	Bartlett et al. (2012)
Capacitance at full turgor	C_ft_	MPa^− 1^	The slope of the relationship between volume and water potential before π_tlp_	Xiong and Nadal (2020)
Osmotic potential at full hydration	π_o_	MPa	Solute concentration in cells at full hydration	Bartlett et al. (2014)
Average root diameter composed the fine root system	Root diameter	mm	Average diameter of root composing fine root system, which relate to soil exploration form	Bergmann et al. (2020)
Specific root length	SRL	m g^− 1^	Root length per dry mass, which indicate the efficiency of soil exploration per unit root mass invested	Freschet et al. (2021)
Root tissue density	RTD	g cm^− 3^	Root dry mass per volume, which relate to root lifespan and mechanical resistance	Freschet et al. (2021)
Root nitrogen content	N content	mg g^− 1^	Amount of nitrogen per root dry mass, which relate to root metabolic activity	Makita et al. (2009)

## Discussion

### Elevational variation in root p-v curve traits

In line with our Hypothesis 1 and Hypothesis 2, the patterns of variation in the p-v curve traits of fine roots were species specific, and only *A. mariesii* showed a decrease in π_tlp_ and C_ft_ with increased elevation from 2,000 to 2,500 m (Fig. 3). Plant cells with a more negative π_tlp_ can maintain turgor and metabolic and growth functions at a more negative Ψ (Bartlett et al. 2012). Moreover, a lower C_ft_ value indicates the ability of plant cells to reduce a volumetric lost as the Ψ declines (Bartlett et al. 2022; Nadal et al. 2018). Thus, the decreases in π_tlp_ and C_ft_ indicate that *A. mariesii* enhances its fine root resistance to water deficit at higher elevations. A previous study reported that *A. mariesii* changed its leaf traits toward a “conservative strategy,” with a lower N content and longer lifespan, along an elevation increase (Takahashi and Miyajima 2008). In this study, the differences in the variation in p-v curve traits of fine roots between *B. ermanii* and *A. mariesii* indicate the existence of a species-specific strategy involving the coordinated resource strategies of the fine roots and leaves for environmental adaptation in subalpine forests.

In contrast to the π_tlp_ and C_ft_ values, the RWC_tlp_ and π_o_ values of fine roots did not change significantly with elevation in either *B. ermanii* or *A. mariesii* (Fig. 3). Variation in π_o_ with elevation was not expected because π_o_ widely varied both across and within species with season and edaphic resource availability in tree leaves, in addition to driving the shift of π_tlp_ (Bartlett et al. 2012; Lenz et al. 2006; Nadal et al. 2023). Moreover, the correlation between π_tlp_ and π_o_ was weaker than that between π_tlp_ and C_ft_ for both *B. ermanii* and *A. mariesii* (Fig. 4). These results suggest that adjusting C_ft_ rather than π_o_ in fine roots is important for tree adaptation to environmental change. The importance of the root C_ft_ in environmental adaptation is supported by previous findings from drought experiments on rootstocks of grapevine seedlings, where a lower root C_ft_ was found to be significantly associated with greater gas exchange in water-stressed plants (Bartlett et al. 2022). A previous study suggested that cell wall stiffness is the main biochemical and structural driver of C_ft_ in leaves, with stiffer walls reducing capacitance by restricting changes in cell volume (Bartlett et al. 2012; Nadal et al. 2018). Therefore, a low C_ft_ may cause mechanical strength in the root (Wang et al. 2021; Ye et al. 2022). For this reason, adjusting π_tlp_ together with C_ft_ would be important in fine roots to maintain the hydraulic function in subalpine cold regions.

In tree leaves, p-v curve traits, particularly π_tlp_, are recognized as key indicators of plant adaptation to drought and show a strong association with water availability within (Bartlett et al. 2014; Mitchell et al. 2008) and across biomes (Bartlett et al. 2012; Zhu et al. 2018). However, we did not find a remarkable difference in the soil Ψ, which reflects plant water availability, between 2,000 and 2,500 m (Table S1). Soil nutrient availability is a possible environmental factor driving changes in the p-v curve traits of fine roots. The total dissolved N content of the soil decreased markedly from 89.6 mg kg^–1^ at 2,000 m to 60.2 mg kg^–1^ at 2,500 m (Table S2). Water loss from root cells causes cell shrinkage and reduces contact between the roots and soil, thus reducing water flow at the soil–root interface (Cuneo et al. 2016; North and Nobel 1997). A reduction in soil-root water flow negatively affects nutrient uptake by the roots, because large quantities of nutrients are dissolved in soil water (Lambers and Oliveiira 2019). Therefore, retention of the root cell volume is important, particularly under nutrient-poor conditions. Although we did not directly measure the root characteristics in winter, given root mechanical damage through compaction and tensile stress, an increase in snowfall and wind at 2,500 m could be a factor in the variation in the p-v curve traits of fine roots (Takahashi et al. 2012). Although the exact environmental factor that drives the change in root p-v curve traits with elevational difference remains unclear, our study suggests that adjusting the root π_tlp_ and C_ft_ would be important for plant adaptation to drought and other abiotic and biotic conditions in subalpine forests. Measuring seasonal variation in the p-v curve traits of fine roots in future research could be useful for detecting the exact environmental factors that drive changes in root p-v curve traits.

### Relationships between the p-v curve traits and morphological and chemical traits of fine roots

Consistent with Hypothesis 3, the root p-v curve traits were related to morphological and chemical traits across elevations, particularly in *A. mariesii*. This suggested the potential coordination of carbon and water economies in fine roots identified in tree leaves (Nadal et al. 2018, 2023; Zhu et al. 2018) (Fig. 6).

In *A. mariesii*, the RTD significantly increased, and the N content significantly decreased with increased elevation from 2,000 to 2,500 m (Fig. 5). Additionally, the RTD correlated negatively with π_tlp_, whereas the N content correlated positively with π_tlp_ and C_ft_, in *A. mariesi* (Fig. 6). These results suggest that there is a specific strategy for *A. mariesii* to adjust p-v curve traits with RTD and N content to acclimate to environmental changes with elevation. Roots with higher RTD typically have longer life spans (Roumet et al. 2016) and lower respiration rates (Makita et al. 2012) than roots with higher N content. Therefore, RTD and N content construct a “conservation gradient” in the root economics space, ranging from the roots with high RTD that show a slow resource return on investment to the roots with high N content for fast resource return on investment (Bergmann et al. 2020; Ding et al. 2023; Makita et al. 2012; Roumet et al. 2016). Our results indicated that water conservation in fine roots is tightly associated with the slow strategy and that roots with a high RTD have high water uptake through a high capacity to preserve root function under stress conditions. This linkage partially explains the relationships between lifespan and the RTD and N content observed in tree fine roots (Freschet et al. 2021; Liu et al. 2016). The RTD was also associated with C_ft_ in *B. ermanii* which did not change any root p-v curve traits or morphological and chemical traits with elevation. This is possibly due to the high variation of the *B. ermanii* traits within the same elevation, suggesting that *B. ermanii* adjusts the C_ft_ of fine roots regardless of elevation. The ability of roots to show strong responses to small-scale environmental changes may have considerable benefits for plant performance in patchy infertile soils especially for disturbance-favoring species like *B. ermanii*. Therefore, more investigations into the variations in p-v curve traits of fine roots at small ecological scales are required to understand the various tree conservation strategies (Weemstra et al. 2021).

### Contrast response in root traits between *B. ermanii* and *A. mariesii*

Our results highlight the contrasting responses of fine root p-v curve traits to elevation between *B. ermanii* and *A. mariesii* (Figs. 3, 5 and 6). In *A. mariesii*, π_tlp_ and C_ft_ decreased from 2,000 to 2,500 m, together with an increase in the RTD and a decrease in the N content. However, no root p-v curve traits or morphological or chemical traits changed with elevation in *B. ermanii*. It has been questioned why the variation in root traits did not differ between elevations only in *B. ermanii*. There are two possible explanations for this observation.

The first is the difference in leaf habits between deciduous and evergreen trees. Regions at higher altitudes typically have shorter growing seasons (Kӧrner 2012). Evergreen plants have thicker leaves with higher leaf mass per area than deciduous species (Reich et al. 1992) and require a longer leaf life span to maximize carbon gain when the favorable period for photosynthesis is shorter because plants cannot afford the cost of constructing new leaves (the cost-benefit model; Kikuzawa et al. 2013). Therefore, *A. mariesii* may need to achieve a longer resource acquisition time even under low nutrient conditions at higher elevations. Conversely, deciduous plants require a shorter leaf life span when the favorable period is shorter because they drop leaves during the unfavorable period. Therefore, *B. ermanii* may need to maximize its ability to maintain the soil-root interface to efficiently obtain resources in a shorter period of time in subalpine regions under short growing seasons and low summer temperatures. Consequently, root traits of *B. ermanii* maintained high values across elevations. Instead, they may adjust the p-v curve traits along the microhabitat at the growth site and optimize root water conservation within the range of their maximum potential (Fig. 5). The less temperature acclimation of *Betula* species was also observed in the root respiration of *B. papyrifera* seedlings (Tjoelker et al. 1999). In deciduous trees, maintaining the physiological function of the fine roots against environmental change may be important for survival in cold regions.

Another aspect is the difference in root morphological traits between broad-leaved and coniferous trees. Among all traits studied, root diameter and SRL strongly explained the difference in fine roots between *B. ermanii* and *A. mariesii* (Fig. 7; MANOVA, *P* < 0.001). Several studies have reported that root diameter and SRL reflect differences in resource acquisition strategies among functional groups and species (Comas and Eissenstat 2009; Eissenstat et al. 2015; Ma et al. 2018; Yahara et al. 2019). Broadleaved species generally have thinner roots and higher SRL values than coniferous species (Comas and Eissenstat 2009; Yahara et al. 2019). These roots have a high soil exploration capacity and high resource uptake efficiency owing to the low cost of root construction (Freschet et al. 2021). Therefore, adjusting water relation traits in fine roots along with large-scale environmental variation might be less important for *B. ermanii* because it can easily reproduce the root or explore and access soil resources. Instead, the adjusting of water relation traits along microhabitat differences at the growth site might be more important for *B. ermanii*; thus, a significant relationship between root p-v curve traits and morphological and chemical traits was observed with elevation (Fig. 5).

Based on the above two aspects, the species-specific patterns of intraspecific variation in p-v curve traits might reflect differences in the capacity of fine roots to resist water deficit at higher elevations between different leaf habits, phylogenies, or combinations of both. Because our study evaluated only two species, the mechanism underlying the contrast response remains unclear. However, our results suggest that leaf habit and/or phylogeny are the key drivers of fine root water relation traits as a basis for water conservation capacity. Further studies with a higher number of elevation levels and/or more species are required to identify broader patterns across species and environmental conditions. This greatly contributes to the understanding of plant-soil interactions in cold regions.

### Conclusion

To the best of our knowledge, this is the first study to have identified species-specific patterns of intraspecific variation in p-v curve traits, particularly π_tlp_ and C_ft_, of fine roots in a subalpine forest. Our findings highlight the importance of fine root traits in acclimation to cold and nutrient-poor subalpine regions. Our findings also identified a species-specific strategy for environmental acclimation through changes in π_tlp_ and C_ft_ driven by RTD and N content. The ability to reduce a volumetric lost as the Ψ decreases and maintain turgor of fine roots is important for accumulation in subalpine cold regions especially for *A. mariesii*, an evergreen conifer. The next step will be to generalize our findings and identify broader patterns across species. Our study of fine-root water relation traits as a basis for water conservation capacity made a breakthrough in furthering our understanding of the strategy for reducing water loss from cells and the cost-benefits between hydraulic efficiencies and carbon utilities through below-ground resource strategies in trees.

## Electronic supplementary material

Below is the link to the electronic supplementary material.

Supplementary Material 1
