# Supplementary material for: Pressure-volume curves of fine roots reveal intraspecific variation across different elevations in a subalpine forest
Source: J Plant Res. 2025 Feb 8;138(3):419–32. doi: 10.1007/s10265-025-01618-8 (PMC12062139; doi:10.1007/s10265-025-01618-8)
Supplement: Supplementary file 1 — Supplementary Material 1 [file 10265_2025_1618_MOESM1_ESM.pdf]

Supporting information

Article title:
Pressure-volume curves of fine roots reveal intraspecific variation across different elevations in a subalpine forest

Journal name:
Journal of Plant Research

Authors:
Taiga Masumoto, Yuki Hashimoto, Takumi Ito, Koichi Takahashi, Naoki Makita*
Faculty of Science, Shinshu University, Matsumoto, Japan
*Correspondence: Naoki Makita, macky@shinshu-u.ac.jp

The following Supporting Information is available for this article:
Fig. S1 Root pressure–volume curve examples from *Betula ermanii* and *Abies mariesii*

Table S1 Air temperature and soil physical properties in 2,000 and 2,500 m during early August to early September. Min, max, and mean values of the daily average at each elevation during the study period are shown for each property

Table S2 Soil chemical properties in 2,000 and 2,500 m

Table S3 Fine root pressure–volume curve traits, morphological and chemical traits of *Betula ermanii* and *Abies mariesii* at 2,000 and 2,500 m

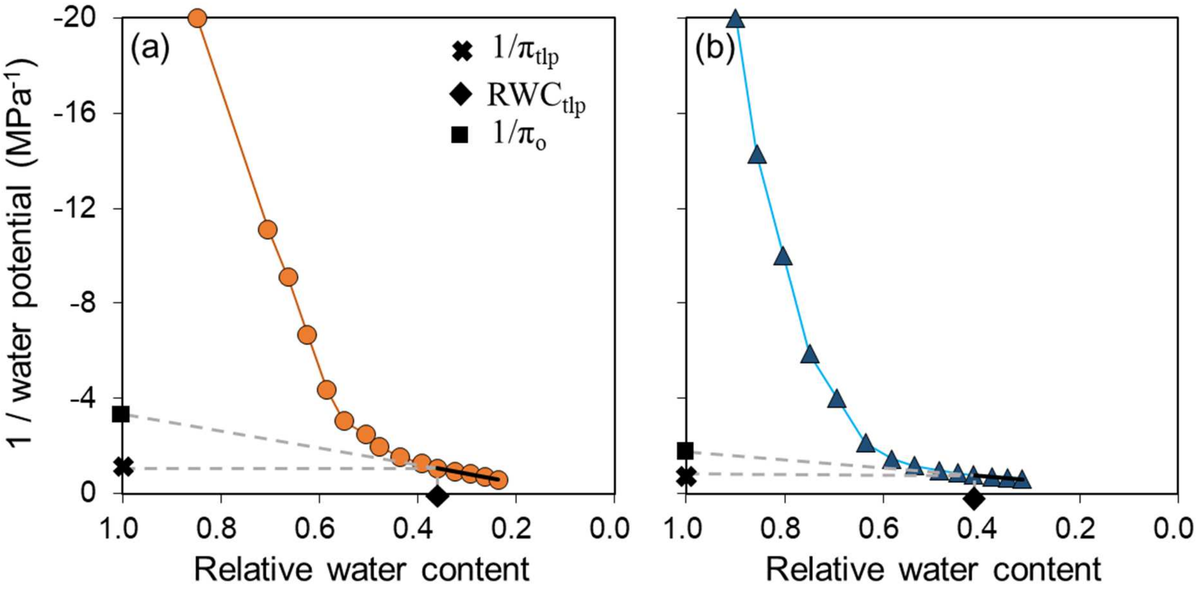

Fig. S1 Root pressure–volume curve examples from *Betula ermanii* (a) and *Abies mariesii* (b). Inverse of turgor loss point ($1/\pi_{tlp}$; cross), relative water content at π_{tlp} (RWC_{tlp}; rhombus), and inverse of osmotic potential at full hydration ($1/\pi_o$; square) are shown. Capacitance (C_{ft}; MPa^{-1}) is defined as the slope of the relationship between relative water content and water potential above π_{tlp}

Table S1 Air temperature and soil physical properties in 2,000 m and 2,500 m during early August to early September. Min, max, and mean values of the daily average at each elevation during the study period are shown for each property

Site elevation	Air temperature [°C]			Soil temperature [°C]			Soil water potential [kPa]		
	Min	Max	Mean	Min	Max	Mean	Min	Max	Mean
2,000 m	12.1	18.7	15.5	12.6	16.3	15.1	−6.34	0	−2.12
2,500 m	9.1	16.6	13.3	10.7	14.7	12.7	−5.56	0	−1.29

Air temperature and soil physical properties at each elevation were monitored using a data logger from early August to early September 2022. Air temperature was monitored at approximately 1.0 m above the ground using a data logger (LR5001, Hioki, Nagano, Japan). Soil physical properties were monitored on the ground at a depth of 0–15 cm using a data logger (Temperature: RC-5, Elitech Technology Inc., CA, USA; water potential: DIK-3210 i Tensiometer, Daiki Rika Kogyo Co., Ltd., Osaka, Japan).

Table S2 Soil chemical properties in 2,000 and 2,500 m. Mean values at each elevation are shown, with standard errors in parentheses (n = 6). NH_4-N, ammonium content; NO_3-N, nitrate content; DON, dissolved organic nitrogen content; TDN, total dissolved nitrogen content; EC, electrical conductivity; C:N, soil carbon to nitrogen ratio.

Site elevation	NH_4-N [mg kg^{-1}]	NO_3-N [mg kg^{-1}]	DON [mg kg^{-1}]	TDN [mg kg^{-1}]	pH	EC [S m^{-1}]	C:N
2,000 m	25.5	0.10	64.0	89.6	4.1	0.009	20.9
	(3.0)	(0.02)	(4.4)	(3.4)	(0.1)	(0.001)	(1.4)
2,500m	20.1	0.17	39.9	60.2	4.2	0.011	21.7
	(3.3)	(0.07)	(7.1)	(10.0)	(0.1)	(0.001)	(2.9)

The chemical characteristics of the soil were measured as follows: Six soil samples were collected from the top 10 cm of the soil below the litter layer at each elevation in September 2022. The samples were sieved through a 2 mm. Dissolved soil nitrogen was measured in fresh samples. The NH_4-N and NO_3-N were measured using the Berthelot and Griess methods, respectively. The TDN content was determined using the Peroxo Oxidizing Reagent method. DON was calculated by subtracting the sum of NH_4-N and NO_3-N from TDN. The samples were then air-dried for two weeks. Soil pH, electrical conductivity (EC), and C:N were measured in dry samples. Soil pH and EC were measured using a portable EC and pH meter (D-210PC, HORIBA, Kyoto, Japan). Total soil C and N contents were determined by dry combustion using an CN analyzer (Flash EA 1112, Thermo Fisher Scientific, Cambridge, MA, USA).

Table S3 Fine root pressure–volume curve traits, morphological and chemical traits of *Betula ermanii* and *Abies mariesii* at 2,000 m and 2,500 m. Mean values for each species at each elevation are shown, with standard errors in parentheses (n = 11).

Species	π_{tlp} [MPa]	RWC_{tlp} [%]	C_{ft} [MPa^{-1}]	π_o [MPa]	Diameter [mm]	SRL [$m\ g^{-1}$]	RTD [$g\ cm^{-3}$]	N content [$mg\ g^{-1}$]
Betula ermanii								
2,000 m	−0.95	38.4	0.63	−0.27	0.49	27.1	0.27	13.7
	(0.09)	(2.4)	(0.05)	(0.02)	(0.02)	(1.8)	(0.02)	(0.96)
2,500m	−1.11	35.4	0.57	−0.32	0.45	33.0	0.26	14.8
	(0.09)	(2.6)	(0.04)	(0.03)	(0.02)	(2.6)	(0.01)	(0.71)
Abies mariesii								
2,000 m	−1.12	48.4	0.41	−0.54	0.71	12.5	0.28	13.4
	(0.04)	(1.9)	(0.01)	(0.02)	(0.02)	(1.1)	(0.01)	(0.48)
2,500 m	−1.46	42.4	0.35	−0.56	0.76	9.7	0.34	9.9
	(0.10)	(2.9)	(0.03)	(0.03)	(0.02)	(1.3)	(0.01)	(0.40)
